# 717 Critically injured patients receiving kefir may have lower rates of Clostridium difficile

**DOI:** 10.1093/jbcr/irac012.271

**Published:** 2022-03-23

**Authors:** Andrea Lillie, Megan Nordlund, Barclay T Stewart, Emma L Gause

**Affiliations:** Harborview Medical Center, Seattle, Washington; Harborview Medical Center, Seattle, Washington; University of Washington, Seattle, Washington; Harborview Medical Center, Seattle, Washington

## Abstract

**Introduction:**

Kefir is an easy to administer per feeding tube probiotic yogurt that does not contain the risk of powdered probiotics, which may contaminate patient wounds or intravenous lines. Previous studies show patients taking probiotics may decrease hospital-acquired infections (HAI) although kefir has not been well studied. We hypothesized that kefir would be well tolerated and prevent infections among critically injured patients including patients with burn injury on enteral nutrition (EN).

**Methods:**

We performed a retrospective review of adult critically injured patients at a level 1 trauma and burn center from January 2018 to March 2021 who received EN. Patients with a history of clostridium difficile (C. diff) were excluded. Patients who received kefir were given 120ml twice daily. The kefir protocol was improved with input from clinical stakeholders. The rate of C. diff, catheter-associated urinary tract infection (CAUTI), and central line-associated blood stream infection (CLABSI) were compared between patients who received kefir and those who did not. Incidence rate ratios (IRR) and corresponding 95% confidence intervals were calculated to assess differences in these rates.

**Results:**

3,814 patients met criteria, 545 of whom received kefir (14%). Suggested improvements to the kefir protocol by stakeholders were changing flavored to plain kefir to decrease the amount of carbohydrate, change to lactose-free kefir to improve usage in lactose intolerant patients, and educate nurses on flushing feeding tubes to avoid clogs.

None of the incidence rates of HAI were significantly different between patients who received kefir and those who did not (Table 1). Crude IRRs suggest that C. diff infections may have occurred less frequently among patients who received kefir while the reverse occurred for CLABSI infections, though these results are not significant.

**Conclusions:**

The kefir implementation was refined by stakeholder feedback. Although no clear benefit of kefir was observed with HAI reduction, future research should investigate the potential association between kefir use and C. diff.

